# A microengineered model of RBC transfusion-induced pulmonary vascular injury

**DOI:** 10.1038/s41598-017-03597-w

**Published:** 2017-06-13

**Authors:** Jeongyun Seo, David Conegliano, Megan Farrell, Minseon Cho, Xueting Ding, Thomas Seykora, Danielle Qing, Nilam S. Mangalmurti, Dongeun Huh

**Affiliations:** 1 Department of Bioengineering, University of Pennsylvania, Philadelphia, PA 19104 USA; 2 Pulmonary, Allergy and Critical Care Division, Perelman School of Medicine, Philadelphia, PA 19104 USA

## Abstract

Red blood cell (RBC) transfusion poses significant risks to critically ill patients by increasing their susceptibility to acute respiratory distress syndrome. While the underlying mechanisms of this life-threatening syndrome remain elusive, studies suggest that RBC-induced microvascular injury in the distal lung plays a central role in the development of lung injury following blood transfusion. Here we present a novel microengineering strategy to model and investigate this key disease process. Specifically, we created a microdevice for culturing primary human lung endothelial cells under physiological flow conditions to recapitulate the morphology and hemodynamic environment of the pulmonary microvascular endothelium *in vivo*. Perfusion of the microengineered vessel with human RBCs resulted in abnormal cytoskeletal rearrangement and release of intracellular molecules associated with regulated necrotic cell death, replicating the characteristics of acute endothelial injury in transfused lungs *in vivo*. Our data also revealed the significant effect of hemodynamic shear stress on RBC-induced microvascular injury. Furthermore, we integrated the microfluidic endothelium with a computer-controlled mechanical stretching system to show that breathing-induced physiological deformation of the pulmonary microvasculature may exacerbate vascular injury during RBC transfusion. Our biomimetic microsystem provides an enabling platform to mechanistically study transfusion-associated pulmonary vascular complications in susceptible patient populations.

## Introduction

Allogeneic RBC transfusions are a lifesaving therapy in perioperative and critically ill patients. Despite its long history and widespread acceptance, transfusion therapy has come under scrutiny due to its association with increased mortality and morbidity in critically ill patients^1–3^. In particular, clinical studies have revealed that RBC transfusions increase the risk of acute respiratory distress syndrome (ARDS) in susceptible patient populations^4, 5^. Although the pathogenesis of this adverse clinical outcome has yet to be elucidated, a mounting body of evidence suggests the pulmonary microvasculature in the distal lung as a key player in transfusion-associated acute respiratory failure^6–8^. For example, transfused RBCs have been shown to activate endothelial cells lining the microvessels in the bronchiolar-alveolar regions and to increase their production of reactive oxygen species and proinflammatory mediators implicated in ARDS^9–11^. As a result of transfusion, pulmonary microvascular endothelial cells can also undergo regulated necrotic cell death termed necroptosis and release immunogenic intracellular molecules known as damage-associated molecular patterns (DAMPs)^7, 12, 13^.

While these findings have improved our fundamental understanding, further progress in this area is hampered by technical challenges of modeling intravascular transfusion of RBCs in the human lung. Current *in vitro* models rely predominantly on static culture of human lung endothelial cells, failing to recapitulate the physiological hemodynamic environment, especially high levels of fluid shear stress in the microvasculature that have a profound influence on endothelial structure and function^14–18^. Moreover, static incubation of RBCs with cultured endothelial cells in these models does not adequately reflect the dynamic interaction of the host vasculature with transfused RBCs *in vivo*. These limitations of *in vitro* techniques have led to widespread use of alternative approaches based on transfusion of living animals or *ex vivo* perfusion of isolated animal lungs^19^. Extrapolation of animal data to human conditions, however, has been highly controversial especially for complex diseases such as ARDS that involve acute injury and inflammatory responses^20^. Consequently, questions remain whether animal models of transfusion are capable of mimicking human-relevant disease processes. The drawbacks of these existing models are emerging as a significant challenge that calls for new strategies to recapitulate the pathophysiology of transfusion-induced vascular complications in the human lung.

**Figure 1:**
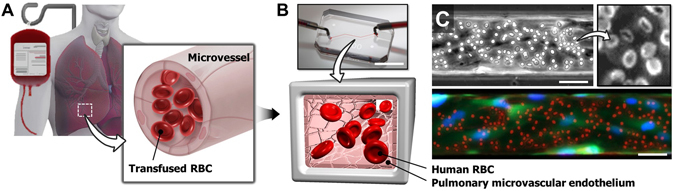
Microphysiological model of RBC transfusion-induced acute vascular injury. (**A**) Blood transfusion-induced vascular injury in the human lung. Transfused red blood cells (RBCs) disperse throughout the lung within microvessels and can cause endothelial injury that often leads to acute respiratory failure in the critically ill. (**B**) The dynamic interaction between transfused RBCs and the pulmonary microvascular endothelium *in vivo* is recreated in a microengineered *in vitro* model consisting of a microfluidic channel lined with primary human lung microvascular endothelial cells. Scale bar: 1 cm. (**C**) The luminal surface of the microfluidic endothelium is perfused with human RBCs to simulate transfusion. In the fluorescence micrograph shown at bottom, endothelial cells and RBCs are stained green and red, respectively. Blue shows nuclear staining in the endothelial cells. Scale bars: 50 μm.

Here we demonstrate the feasibility of leveraging a microengineered cell culture platform to tackle this critical challenge. Specifically, we describe a specialized *in vitro* model to replicate i) the native phenotype and hemodynamic environment of the pulmonary microvascular endothelium and ii) physiologically relevant endothelial interaction with transfused allogeneic RBCs in the human lung (Fig. 1A). This microphysiological model is established by forming a perfusable vascular lumen lined with primary human pulmonary microvascular endothelial cells in a simple microfluidic channel that approximates the size of microvessels in the human lung. The intraluminal compartment of this model is perfused at physiological levels of shear stress to mimic hemodynamic flow and RBC transfusion *in vivo* (Fig. 1B,C). Using this microsystem, we investigated deleterious effects of RBCs on the lung microvascular endothelium during transfusion. Our study demonstrated that RBC transfusion induces DAMP release associated with necroptosis of endothelial cells and leads to acute vascular injury consistent with previous *in vivo* findings. This adverse response was accompanied by aberrant alterations of intracellular structures in the vascular endothelium. We also discovered that fluid shear stress generated by intravascular flow is an important determinant of transfusion-induced endothelial injury. Moreover, we further engineered our model to expose the cultured endothelial cells to both hemodynamic shear stress and cyclic mechanical stretch reminiscent of breathing-induced vascular tissue deformation during RBC transfusion. Data from this combined model showed that physiological mechanical forces generated by cyclic breathing motions may aggravate the injurious effects of transfused RBCs on the pulmonary microvasculature.

Our vascular injury-on-a-chip provides an example of a minimalist approach to the development of predictive human disease models which are both clinically and physiologically relevant. This system may serve as a basis for creating a novel research platform to investigate the mechanisms of respiratory complications following blood transfusion.

## Results and Discussion

### Formation of lung microvascular endothelium

**Figure 2:**
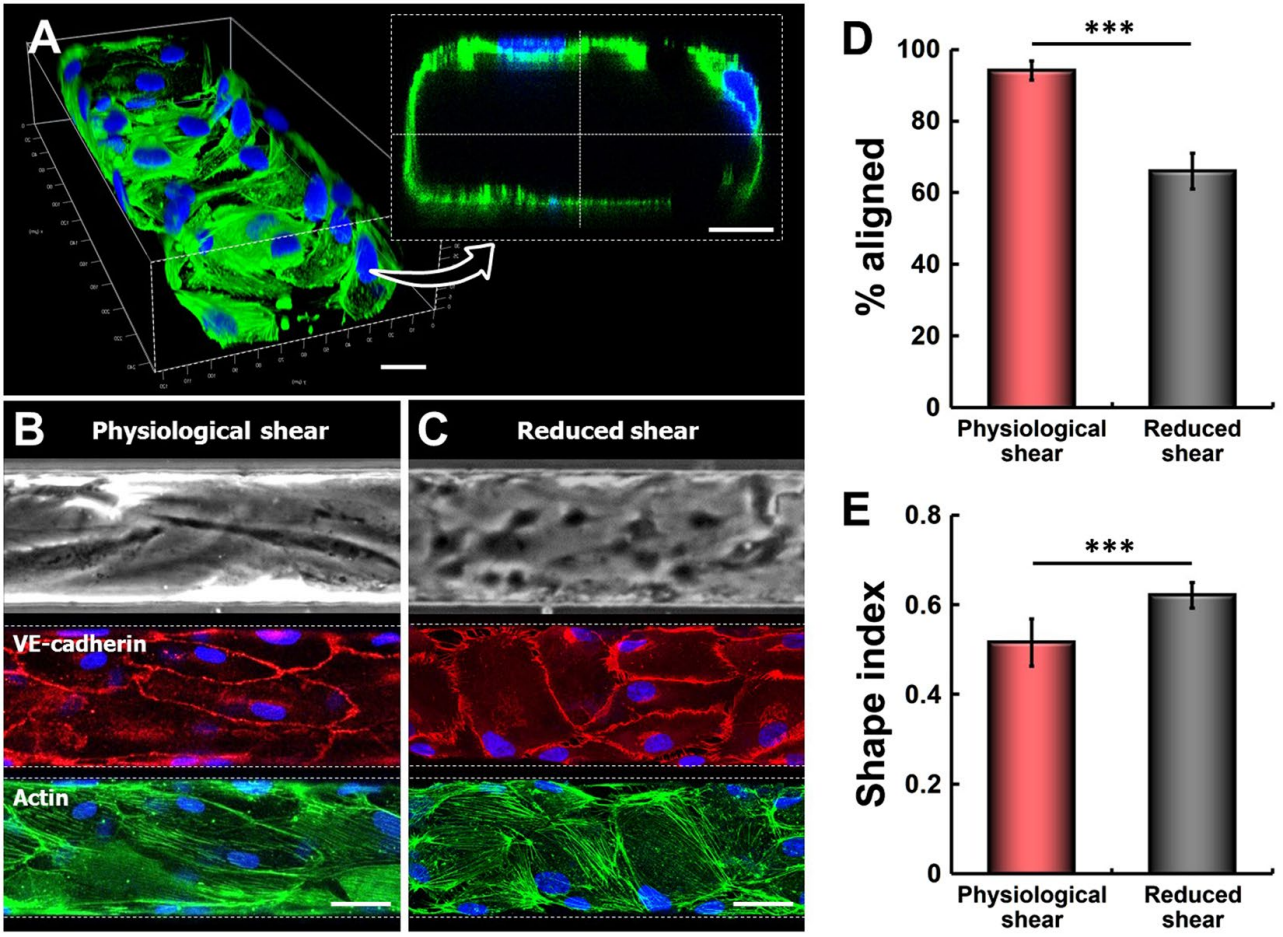
Formation of the microengineered endothelium with physiological endothelial phenotypes. (**A**) Primary human lung microvascular endothelial cells seeded into the microchannel at high densities attach to the top and bottom channel surfaces and the sidewalls. The attached cells begin to spread rapidly. The images show fluorescently labeled cells at 1 hour after seeding. Scale bars: 20 μm. (**B**) During growth, the cultured endothelial cells become elongated and reorient themselves in the direction of flow. Perfusion culture for 4 days results in the formation of a confluent endothelial monolayer that expresses adherens junctions (VE-cadherin, red) and highly organized actin cytoskeleton (F-actin, green). Nuclei are stained with DAPI (blue). Physiological shear refers to 14.8 dyn/cm^2^. (**C**) In contrast, cell elongation and alignment become much less evident at reduced shear stress (1.78 dyn/cm^2^). The images were taken at day 4. (**D**) The faction of aligned endothelial cells at physiological shear stress (14.8 dyn/cm^2^) is significantly larger than that at reduced shear stress (1.78 dyn/cm^2^) (***p < 0.001; n = 4). (**E**) Physiological shear stress also increases cytoplasmic elongation of the cultured cells (***p < 0.001; n = 4). Scale bars: 50 μm.

Following seeding into the microchannel, endothelial cells established firm adhesion to the ECM-coated channel walls and began to spread within 1 hour under static conditions. Combined with the small dimensions of the channel, the high cell seeding density used in our experiments allowed the seeded cells to attach not only to the bottom surface but also to the vertical sidewalls and ceiling of the channel. After initial attachment, the cells were observed to conformally cover the microchannel surfaces and form an enclosed lumen structure with a rectangular cross-section (Fig. 2A). At the sharp corners of the channel, however, many of the cells failed to show the same extent of conformal adhesion and often formed an arch between two neighboring channel walls, making the corners of the microfluidic endothelial lumen rounded (inset, Fig. 2A). When the attached cells were perfused with culture medium, they remained adherent and increased their spreading in spite of physiologically relevant high shear stress exerted on the channel walls. During perfusion culture, the cells were observed to proliferate continuously over a few days until full confluence was reached. As shown in Fig. 2B, immunofluorescence analysis revealed a tightly packed microvascular endothelium with robust expression of adherens junctions, such as VE-cadherin, throughout the microchannel. The junctions were clearly visible along the borders between adjacent cells, and no intercellular gaps were detected. Structural integrity of the endothelium was also evident from staining of actin cytoskeleton (Fig. 2B). Moreover, the vast majority of the cells showed cytoplasmic elongation and linear assembly of stress fibers along the longitudinal direction of the channel, closely resembling the characteristic morphology of the vascular endothelium *in vivo* ^21^.

### Recapitulation of native endothelial phenotypes

Vascular endothelial cells sense their hemodynamic microenvironment and respond to fluid shear stress produced by blood flow in the vascular lumen^16, 22^. Endothelial alignment is a well-documented example of such responses that involves elongation and reorientation of endothelial cells in the direction of blood flow^23, 24^. The ability of our microengineered model to generate and control media flow during perfusion culture provided a means to reconstitute these physiologically relevant morphological changes.

In response to intraluminal flow that generated physiological levels of wall shear stress (14.8 dyn/cm^2^), many of the endothelial cells showed significantly elongated cytoplasm extending preferentially in the direction of flow (Fig. 2B). Quantitative analysis of the orientation angle indicated that nearly 95% of the cells exhibited similar morphological responses (Fig. 2D). This flow-induced reorientation of cytoplasm and intracellular structures, however, became significantly less evident when the cells were cultured at reduced shear stress (1.78 dyn/cm^2^) consistent with the values used in previous studies to simulate sub-physiological hemodynamic stress^22, 25^ (Fig. 2C). In this case, the fraction of aligned cells decreased to approximately 65% (*p < 0.05) (Fig. 2D). Under this condition, the non-aligned cell population remained randomly oriented regardless of the duration of perfusion culture. Evaluation of the shape index (SI) also provided quantitative evidence that the fluid dynamic microenvironment has a significant influence on cell shape in our model. As illustrated in Fig. 2E, treatment of the endothelium with physiological shear stress led to cytoplasmic elongation of the endothelial cells and resultant reduction in the SI as compared to that measured at lower shear stress (*p < 0.05). These results demonstrate the significance of physiological flow conditions as a critical requirement for recapitulating native morphological phenotypes of the vascular endothelium.

### Acute vascular injury due to RBC transfusion

Using the physiological microfluidic endothelium, we then investigated whether our model was capable of reconstituting transfusion-induced acute vascular injury *in vivo*. A primary focus of this study was on the analysis of HMGB1 in the cultured endothelial cells. HMGB1 is a DNA-binding protein in the nucleus that serves to stabilize nucleosome formation and to regulate expression of several genes^26^. During necroptosis, however, nuclear HMGB1 is released into the extracellular space and acts as a cytokine that mediates immune cell activation and systemic inflammation^27^. Recent studies have suggested this cell death pathway as a novel mechanism of vascular injury and inflammation induced by transfused RBCs^7^.

**Figure 3:**
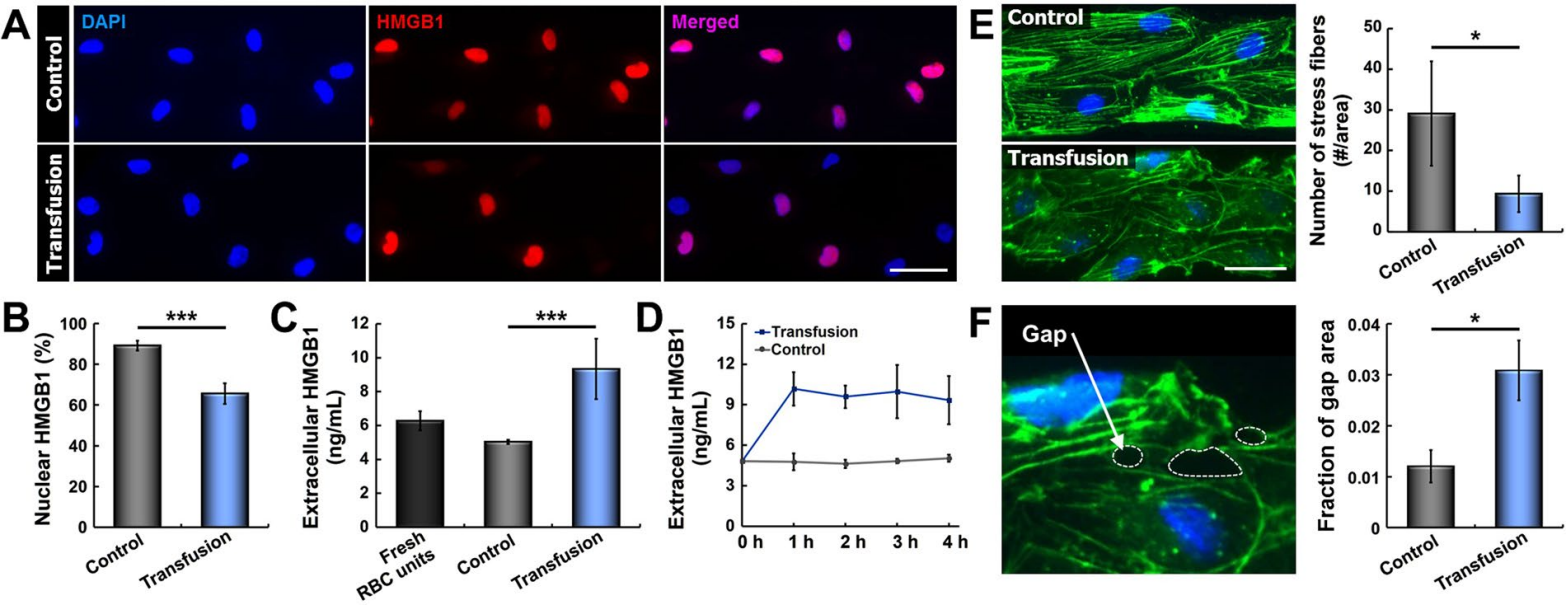
RBC transfusion-induced acute vascular injury. (**A**) Continuous RBC transfusion for 4 hours results in a loss of nuclear HMGB1 (red). Control data show HMGB1 expression in endothelial cells perfused with culture medium alone for the same period. Nuclei are stained with DAPI (blue). (**B**) Quantification of nuclear HMGB1 staining under control (without RBCs) and transfusion conditions (***p < 0.001, n = 4). (**C**) Quantification of extracellular HMGB1 measured in the fresh RBC units and perfusate collected from the control and transfusion devices (***p < 0.001; n = 4). (**D**) Extracellular HMGB1 released from the RBC-perfused microfluidic endothelium significantly increases within the first hour of transfusion, whereas the HMGB1 levels remain low and nearly constant in the control group. (**E**) Exposure of the endothelial cells to RBCs also leads to disorganization of cytoskeletal actin filaments and significant reduction in the number of stress fibers (*p < 0.05; n = 3). (**F**) The deleterious effects of RBC transfusion also include the formation of intercellular gaps indicated with white dotted lines in the fluorescence micrograph. Green and blue show F-actin and nuclei, respectively. As compared to control, perfused RBCs significantly increase the surface area of intercellular gaps in the endothelium (*p < 0.05; n = 3). Scale bars: 50 μm.

Based on these findings, we examined changes in the levels of intracellular and extracellular HMGB1 due to microfluidic RBC transfusion in our model. When the endothelium was perfused with RBCs for 4 hours under physiological flow conditions, the vast majority of the cells remained adherent without any indication of endothelial denudation. Immunofluorescence analysis of HMGB1, however, revealed significant detrimental effects of RBC transfusion. In comparison to the control group treated with acellular culture medium, the cells perfused with RBCs showed approximately 20% reduction in HMGB1 staining in their nuclei (Fig. 3A,B). Although the extent of decrease in immunofluorescence varied slightly from cell to cell, this acute response occurred uniformly throughout the entire endothelium. To investigate if this significant loss of nuclear HMGB1 was due to extracellular release of HMGB1 caused by necroptotic cell death in the vascular endothelium, we analyzed the level of HMGB1 detected in the transfused RBCs and culture medium collected from the channel outlet. As shown in Fig. 3C, the concentration of extracellular HMGB1 measured in the transfusion model was nearly 50% higher than that in the fresh RBC units prior to transfusion experiments and the control group perfused with culture medium alone (i.e. without RBCs). This significant increase took place within the first hour of transfusion, and the HMGB1 levels remained high throughout transfusion (Fig. 3D). Considering that RBCs are an unlikely source of HMGB1 due to the absence of nucleus^28^, these data suggest that RBC transfusion plays a causative role in the development of acute vascular injury in our model by triggering necroptosis of lung endothelial cells, as verified by the increased endothelial release of nuclear HMGB1 into the extracellular compartment. This observation is consistent with previous *in vivo* findings that transfused RBCs induce lung necroptosis and increase the levels of HMGB1 circulating in the plasma^9^, demonstrating the physiological relevance of our microfluidic model.

To further characterize adverse effects of RBC transfusion, we carried out microfluorimetric analysis of actin cytoskeleton in the microengineered vascular endothelium. When the cells were subjected to continuous flow of culture medium in the absence of RBCs, they displayed highly organized cytoskeletal architecture evident from well-defined actin stress fibers across the cell body that aligned in the direction of fluid flow (Fig. 3E). Inclusion of RBCs in the perfusate, however, resulted in considerable loss of stress fibers in the cytoplasm, which was concurrent with organization and concentration of actin filaments along the cell periphery (*p < 0.05) (Fig. 3E). Interestingly, this cytoskeletal rearrangement was accompanied by the formation of intercellular gaps throughout the endothelium (*p < 0.05) (Fig. 3F). This finding is particularly significant in that it demonstrates the capacity of transfused RBCs to compromise the structural integrity of the pulmonary vascular endothelium. In fact, studies have shown that damage-associated signals such as HMGB1 released from injured endothelial cells can increase vascular permeability and induce endothelial barrier dysfunction during acute lung injury^29, 30^. Therefore, we suspect that paracellular gap formation in our microfluidic endothelium is mediated by extracellular HMGB1 released from the endothelial cells undergoing necroptosis during RBC transfusion.

### Effect of hemodynamic shear stress on transfusion-induced injury

Blood flow is essential to the physiological microenvironment of vascular endothelial cells. In particular, fluid shear stress exerted by hemodynamic flow on the blood vessel wall plays a critical role in vascular homeostasis by modulating the structure and function of endothelial cells^15, 22, 31^. Sustained alterations in wall shear stress often lead to endothelial dysfunction that contributes to the pathogenesis of various vascular diseases^25^. For example, when blood flow is disturbed due to arterial bifurcations, low and oscillatory shear stress in regions of flow disturbance has been shown to activate inflammatory signaling pathways in vascular endothelial cells that promote the development of atherosclerosis^22, 32, 33^. In contrast, high and laminar shear stress is protective against atherogenesis by upregulating expression of endothelial genes responsible for suppressing inflammation and oxidative stress^25^. Based on this well-established importance of hemodynamic forces, we asked whether fluid shear stress had a significant influence on transfusion-induced endothelial injury in our model.

**Figure 4:**
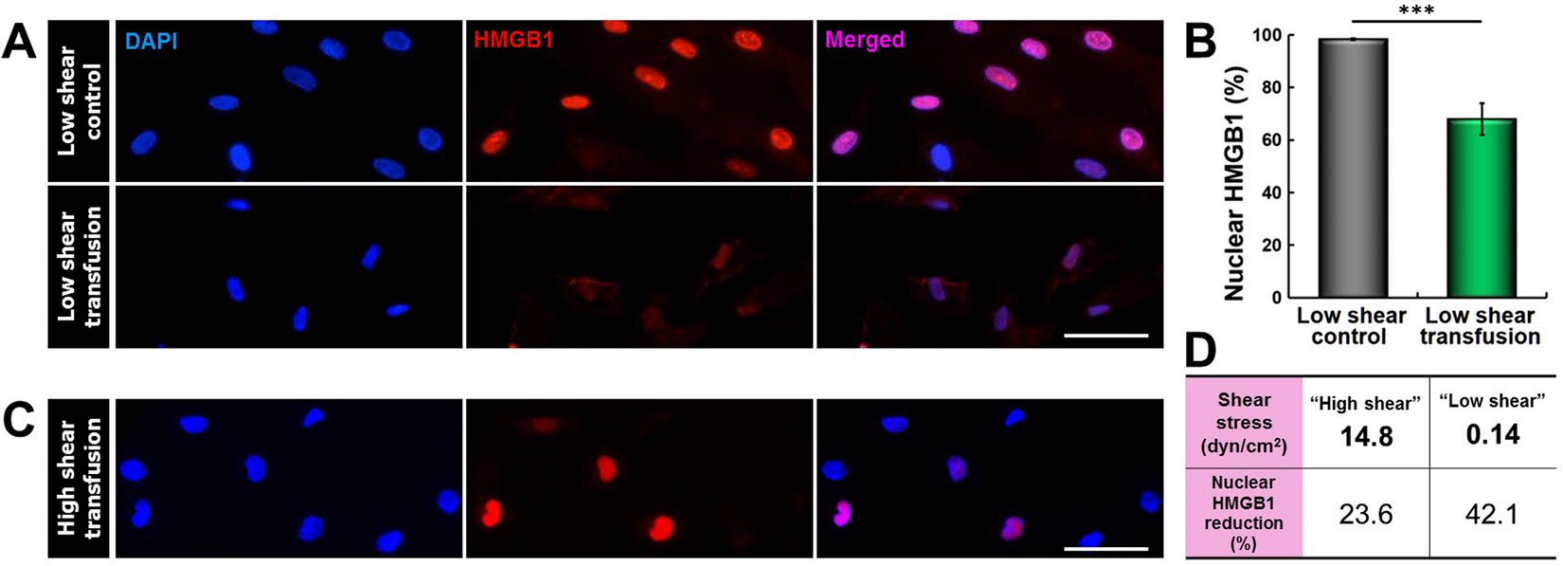
Effect of hemodynamic shear stress on transfusion-induced endothelial injury. (**A**,**B**) Endothelial perfusion with RBCs at a sub-physiological level of shear stress (0.14 dyn/cm^2^) gives rise to marked downregulation of nuclear HMGB1 expression (“low shear transfusion”), indicating severe necroptosis in the microvascular endothelium (***p < 0.001; n = 3). “Low shear control” refers to perfusion of the microfluidic endothelium at 0.14 dyn/cm^2^ with culture medium alone. Blue shows nuclear staining. (**C**,**D**) Qualitative and quantitative comparisons of transfusion-induced nuclear HMGB1 loss between the sub-physiological “low shear” (0.14 dyn/cm^2^) and physiological “high shear” (14.8 dyn/cm^2^) conditions. RBC perfusion at physiological shear stress results in significantly smaller HMGB1 reduction, suggesting the protective role of physiological hemodynamic microenvironment. Scale bars: 50 μm.

To examine this question, we carried out microfluidic culture and RBC perfusion at substantially reduced shear stress (“low shear”, 0.14 dyn/cm^2^) and analyzed resultant DAMP release. The magnitude of the applied shear stress was estimated based on previous reports that pathological hemodynamic conditions in susceptible patient populations can lead to more than two orders of magnitude reduction in wall shear stress as compared to normal subjects^25^. Endothelial exposure to RBCs in this altered hemodynamic environment led to pronounced loss of intracellular HMGB1 (“low shear transfusion” in Fig. 4A). Compared to the control group perfused at the same low shear stress without RBCs (“low shear control” in Fig. 4A), quantitative evaluation showed a decrease of roughly 40% in the average intensity of nuclear HMGB1 in the transfused devices (***p < 0.001) (Fig. 4B). Interestingly, this reduction of nuclear HMGB1 in the “low shear” conditions was significantly greater than that measured in the physiologically relevant flow conditions (“high shear”, 14.8 dyn/cm^2^) (Fig. 4C,D). These results illustrate that reduced shear stress increases the detrimental effect of transfused RBCs and induces more severe vascular injury. Importantly, this finding is supported by clinical reports that critically ill patients with increased susceptibility to transfusion-related injuries (e.g., septic patients) often display persistent microvascular alterations characterized by significantly reduced blood flow velocity^34^. Evidence has also demonstrated that increased thrombosis and impaired vasodilation in the microvasculature of these patients can lead to microcirculatory hypoperfusion and concomitant reduction in hemodynamic forces^35^.

In light of these observations, our study suggests that lower hemodynamic shear stress due to altered microcirculatory flow in the critically ill may predispose lung endothelial cells to necroptosis and exacerbate their adverse responses to transfused RBCs. This condition will likely elevate the severity of vascular injury and inflammation in the lung, increasing the susceptibility of transfused patients to respiratory complications. Conversely, our data also imply that the physiological hemodynamic microenvironment may have a protective role in transfusion-induced pulmonary vascular injury. As evidenced by this potential disease mechanism, compromised hemodynamic homeostasis may play a critical role that has largely been overlooked in the traditional study of transfusion-associated ARDS. For *in vitro* studies, these new insights into the importance of hemodynamics provides a strong justification for the development of dynamic cell culture systems to accurately model and predict adverse impact of RBC transfusion.

### Effect of mechanical stretch on transfusion-induced vascular injury

Expansion and contraction of the air sacs during respiration induce cyclic mechanical stretch of the microvasculature in the alveolar regions of the lung^36, 37^. Along with hemodynamic shear stress, this type of solid mechanical stress generated by breathing motions plays an important role in the structure and function of lung endothelial cells^24, 36, 37^. In particular, studies have shown that breathing-induced physical forces significantly influence the response of the pulmonary microvascular endothelium to inflammatory insults during the development and progression of acute lung injury^38–40^. Based on these findings, we questioned whether and how physiological mechanical stretch of endothelial cells affected their injury response to transfused RBCs in our model.

**Figure 5:**
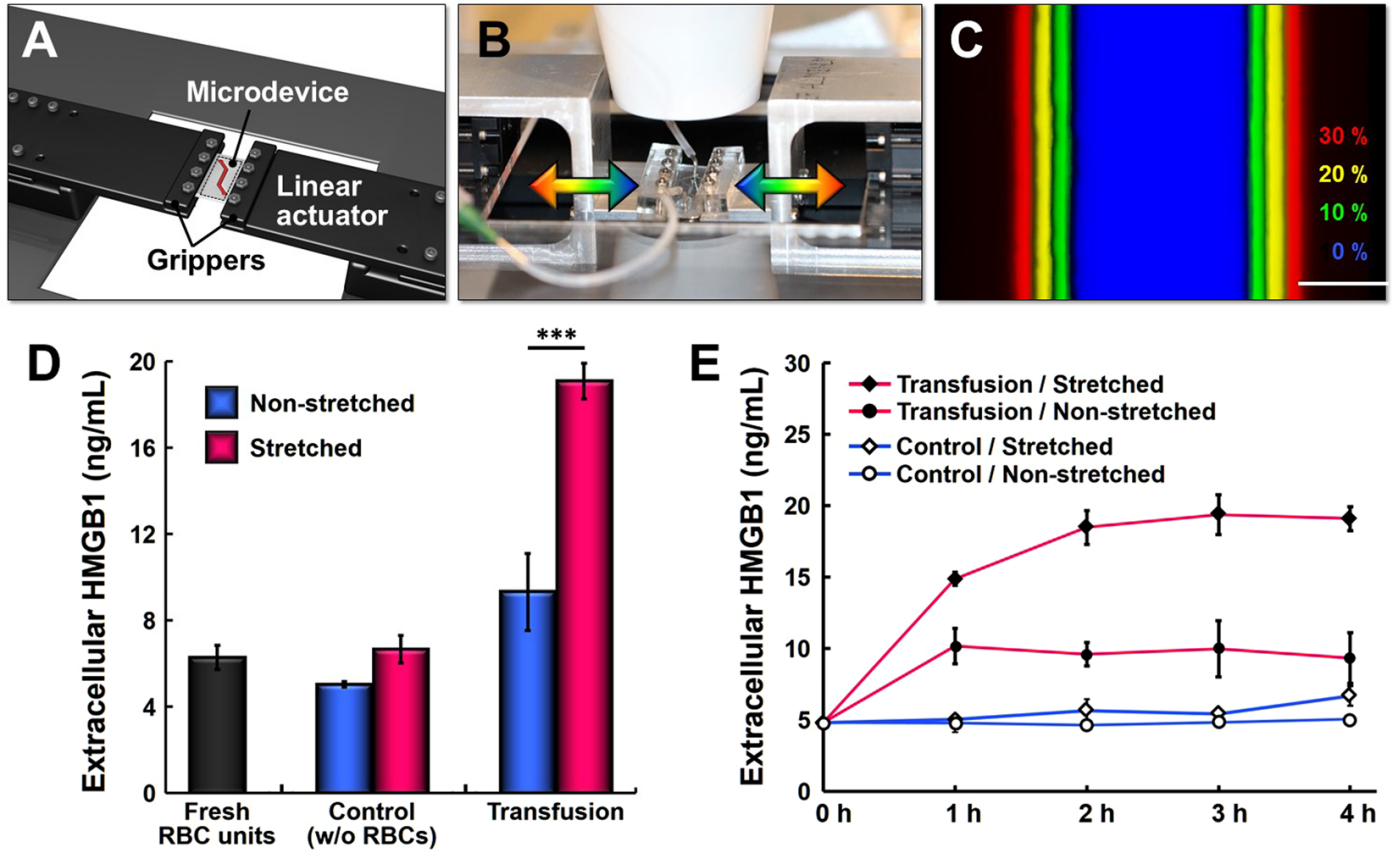
Effect of mechanical stretch on transfusion-induced vascular injury. (**A**) The cell stretching system consists of grippers and motorized linear actuators that hold and stretch our cell culture microfluidic device in the lateral direction during perfusion experiments. (**B**) The entire platform can be mounted on a microscope stage for real-time monitoring and imaging of our model. (**C**) Uni-axial stretching of the microchannel by computer-controlled stretching system (Scale bar: 50 μm). The same microchannel was pseudo-colored at different levels of strain to show stretch-induced widening of the channel. (**D**) Quantification of extracellular HMGB1 measured in the fresh RBC units and perfusate from the control and transfusion groups in the presence and absence of cyclic stretch. Increases in the level of extracellular HMGB1 due to RBC transfusion are substantially higher when the endothelium is mechanically stretched at physiological strain (10%) and frequency (0.2 Hz) (***p < 0.001; n = 4 and n = 2 for the non-stretched and stretched groups, respectively). (**E**) Time-course measurement of extracellular HMGB1 in the tested groups. The concentration of extracellular HMGB1 in the stretched transfusion group (closed diamond) significantly increases within the first two hours of RBC transfusion. The rate of HMGB1 increase in this group is greater than that in the non-stretched transfusion group (closed circle).

As the first step to examine this question, we tested our custom-built cell stretching system shown in Fig. 5A,B. The design of this platform made it possible to mount the entire stretching unit on a microscope stage and to stably hold our perfused microdevice during stretch when real-time monitoring and visualization were necessary (Fig. 5B). The computer-controlled operation of this system permitted precise application of varying levels of uniaxial strain across the width of the channel (Fig. 5C). To simulate physiologically relevant conditions, we applied 10% strain at the frequency of normal breathing or mechanical ventilation (0.2 Hz)^41^. Using this system, we first carried out control experiments to measure extracellular release of HMGB1 from endothelial cells perfused with culture medium alone (i.e. without RBCs) in the presence and absence of cyclic strain. Our results yielded no significant difference between the two groups (Fig. 5D), providing evidence consistent with previous findings that physiological mechanical stretch itself does not exert deleterious effects on endothelial cells^36, 37^. When the cells were perfused with RBCs, however, the applied mechanical strain was found to be an important determinant of the extent of endothelial injury due to transfused RBCs. Specifically, application of 10% physiological strain at 0.2 Hz during microfluidic RBC transfusion led to a more than two-fold increase in extracellular release of HMGB1, as compared to the production of the same damage signal measured in the transfused endothelium without stretching (Fig. 5D). Our time-course measurement showed that significant injury responses of the stretched endothelium occurred in the first two hours of transfusion, after which the increased levels of HMGB1 release were maintained over the duration of RBC perfusion (Fig. 5E). This rapid increase in HMGB1 release illustrates the acute nature of endothelial injury in our model and also correlates with the clinical observation that transfusion-induced acute lung injury usually develops within the first five to six hours of transfusion^42–44^. More importantly, these data suggest that cyclic stretching of the lung microvessels due to physiological breathing movements or mechanical ventilation may increase the susceptibility of the pulmonary endothelium to transfused RBCs and contribute to the development of vascular injury in the lung.

## Conclusion

This paper demonstrates the feasibility of engineering a microphysiological model that captures the essential disease process of RBC transfusion-induced acute vascular injury in the lung. Despite its simple design and operation, this microengineered device provides an enabling platform to simulate and examine endothelial dysfunction central to the life-threatening complications of blood transfusion that remain clinically significant yet poorly understood. In the United States alone, nearly 14 million blood units are transfused annually^45^. Considering that the safety and beneficial effect of this common clinical practice now face increased scrutiny^46^, our study is timely and may offer opportunities to develop new strategies for systematically investigating injurious effects of transfusion.

While our microengineered system enables capabilities not possible in traditional static cell culture models, improvements can be made to more faithfully recapitulate the native environment of the microvasculature in transfused lungs. For example, our system may benefit from the ability to model the interaction of the vascular endothelium with blood borne immune cells. Although leukoreduced RBC units have gained widespread use, residual leukocytes in the transfusate can be activated by HMGB1 released from necroptotic endothelial cells and secrete various cytokines that may aggravate endothelial injury and trigger inflammatory responses. Pro-inflammatory mediators circulating in the blood are another potential contributor to vascular complications of transfusion therapy that has yet to be incorporated into our model. High systemic levels of inflammatory cytokines are common in critically ill patients and may render endothelial cells more susceptible to injury, increasing the deleterious effects of transfused RBCs. For instance, studies have shown that endothelial activation due to pro-inflammatory insults increases adhesion of transfused RBCs to the microvasculature^47^, which can lead to impaired tissue perfusion and increased vascular injury. This type of disease microenvironment can be recapitulated in our model to examine how inflammatory stimuli contribute to endothelial injury in transfused patients. Finally, further engineering of our microdevice is required to establish more physiological models capable of simulating the cross-talk between the pulmonary vascular endothelium and the alveolar epithelium. This type of systems would make it possible to mimic key pathologies of the alveolar-capillary unit directly responsible for post-transfusion lung injury and acute respiratory failure.

From a more general perspective, this study lays the groundwork for the development of innovative technologies to investigate blood-tissue interactions essential to the physiology of the human body. The dynamic cell culture system demonstrated in this work may also serve as a technical basis for creating a clinical screening platform to detect potentially adverse effects of blood transfusion. Considering the recent finding that lung microvascular endothelial cells undergo necroptosis following direct contact with circulating tumor cells^48^, cancer research is another area that may immediately benefit from the demonstrated capabilities of our platform. We believe that our microengineering strategy holds great potential as a new frontier in transfusion medicine and many other related fields.

## Methods

### Cell culture and RBC preparation

Primary human lung microvascular endothelial cells (HMVEC-L) were obtained from Lonza (CC-2527, Basel, Switzerland) and cultured in endothelial growth medium supplemented with hEGF, hydrocortisone, Gentamicin, Amphotericin-B, 5% FBS, VEGF, hFGF-B, R3-IGF-1, and ascorbic acid (EGM-2 MV, CC-3202, Lonza). Leukoreduced human RBC units were obtained from the blood bank at the Hospital of the University of Pennsylvania and rinsed by centrifugation with sterile PBS before use.

### Microdevice fabrication

Soft lithography was used to fabricate microfluidic devices for cell culture. Briefly, the upper layer of the microfluidic device was produced by casting poly(dimethylsiloxane) (PDMS) against a photolithographically-prepared master that contained a positive relief pattern of a microchannel made of photoresist (SU-8, MicroChem). PDMS pre-polymer (Sylgard 184 Silicone elastomer kit, Dow Corning, Midland, MI) was thoroughly mixed with a curing agent at a weight ratio of 10:1 (PDMS:curing agent) and poured onto the microfabricated master. After curing at 65 °C overnight, the channel slab was removed from the master. The cross-sectional dimensions of the channel were 100 μm (width) by 50 μm (height). The bottom layer of the device was prepared by pouring a mixture of PDMS pre-polymer and curing agent into a 15 mm Petri dish and curing it at 65 °C overnight. For bonding, PDMS pre-polymer was mixed with curing agent at a weight ratio of 10:3 and spin-coated on a 100 mm Petri dish at 9000 rpm for 5 minutes. Subsequently, the channel layer was gently placed on the dish to form a thin film of uncured PDMS on the surface of the PDMS slab. The channel slab was then bonded to the bottom substrate to create an enclosed microchannel. The assembled device was incubated at 65 °C overnight to ensure complete bonding.

### Microfluidic cell culture

The fully assembled microdevices were sterilized by UV irradiation (UVLS-26, EL Series Six Watt Lamps, 2UV 365 nm/254 nm, Upland, CA) for 30 minutes prior to cell culture. For cell adhesion, the devices were incubated with a fibronectin solution (40 μg/mL) (356008, Fibronectin, human, 5 mg, BD Biosciences, San Jose, CA) overnight. Subsequently, lung endothelial cells suspended in their culture medium at a concentration of 2 × 10^7^ cells/mL were injected into the microchannel and allowed to attach to the fibronectin-coated channel surfaces over a period of 1 hour under static conditions. Once cell attachment was established, a continuous flow of culture medium was generated in the microchannel by a syringe pump (BS 8000, 120 V, Braintree Scientific, Inc., Braintree, MA). The device was perfused at a volumetric flow rate of 250 μL/h for at least 3 days prior to RBC perfusion. Wall shear stress at this flow rate was evaluated to be 14.8 dyn/cm^2^, closely matching the mean wall shear stress (15.4 dyn/cm^2^) measured in the pulmonary microvessels of the human lung^49^. Since the pulsatility of blood flow due to the rhythmic contraction of the heart is negligible in the microvessels of the distal lung, we used steady flow conditions in our experiments^50–52^.

### Visualization of the microfluidic endothelium

We performed immunofluorescence staining of F-actin and VE-cadherin expressed by the microengineered endothelium to demonstrate the formation of physiological vascular tissue in our model. As the first step of this procedure, the cells were fixed with a 4% paraformaldehyde solution for 15 min, and the reversibly bonded microchannel layer was removed from the microdevice to gain direct access to the cells on the bottom channel surface. The cells were then permeabilized with 0.5% Triton X-100 for 15 min. After washing, the cells were incubated with Alexa Fluor 488 conjugated Phalloidin (A12379, Life Technologies, Carlsbad, CA) and DAPI for 20 min to stain F-actin and nuclei, respectively. For visualization of adherens junctions, the cells were first incubated overnight with primary VE-cadherin antibody (2500 S, Cell Signaling, Danvers, MA) diluted in 2% BSA solution. Secondary anti-rabbit antibody conjugated to Alexa Fluor 594 (ab150080, Abcam, Cambridge, MA) was diluted in 2% BSA solution and incubated for 1 hour. Nuclei were labeled with DAPI. Images of the fluorescently stained cells were acquired by confocal microscopy (Leica, Wetzlar, Germany).

### Quantitative analysis of endothelial responses to shear stress

To investigate the response of the microfluidic endothelium to physiological shear stress, we carried out image analysis of cell morphology and evaluated the Shape Index (SI) defined by the following equation^23^,

$$SI=\,4\pi \frac{A}{{p}^{2}}$$

where *A* and *p* are the area and perimeter of the cell, respectively.

Characterization of morphological responses to hemodynamic forces was also accomplished by analyzing flow-induced reorientation and alignment of the cultured endothelial cells. Specifically, cell alignment was quantified by measuring the positive angle between the major axis of the cell body and the vertical axis. The cell was considered aligned when this angle lied between 60 and 120 degrees.

### Microfluidic RBC transfusion

Based on previous studies^11^, the microengineered endothelium was perfused with endothelial growth medium containing 20% (v/v) human RBCs to simulate RBC transfusion. Microfluidic RBC perfusion was maintained at 250 μL/h for 4 hours to reconstitute physiological hemodynamic shear stress and to match the duration of transfusion that has been shown clinically to induce significant vascular responses *in vivo* ^7^.

### Characterization of vascular injury due to RBC transfusion

Studies have shown that banked RBCs can induce necroptosis of vascular endothelial cells during transfusion, leading to cellular release of nuclear proteins such as HMGB1 into the extracellular space^7^. Therefore, we characterized deleterious responses of our model to RBC transfusion by measuring i) a loss of HMGB1 in the nuclei of transfused endothelial cells and ii) the concentration of released extracellular HMGB1 detected in the perfused RBCs and culture media. For analysis of nuclear HMGB1, immunofluorescence techniques were used. Briefly, the endothelial layer was first rinsed with DPBS after transfusion to remove remaining RBCs and culture medium. After the microchannel was peeled off, the cells lining the bottom substrate were fixed and permeabilized using the same methods described above. The endothelium was then incubated with primary rabbit polyclonal anti-HMGB1 antibody (ab18256, 5 μg/mL, Abcam) overnight. Subsequently, the cells were washed and treated with secondary anti-rabbit antibody conjugated to Alexa Fluor 594 (ab150076, 5 μg/mL, Abcam) for 45 min. Nuclei of the same cells were stained with DAPI. After a final washing step, the labeled cells were imaged using an epifluorescence microscope (Carl Zeiss, Oberkochen, Germany). For analysis of intracellular HMGB1, co-localization of HMGB1 and DAPI staining was evaluated quantitatively using ImageJ. To evaluate actual release of HMGB1 from the endothelial cells into the extracellular space, the perfused RBCs and culture media were collected every hour over the course of transfusion experiments. The concentration of HMGB1 in the collected samples was measured using a commercial HMGB1 detection kit following manufacturer’s protocol (Chondrex, Redmond, WA).

To analyze the formation of intercellular gaps due to RBC transfusion, F-actin and cell nuclei were stained for immunofluorescence using the protocol described above (see “Visualization of the microfluidic endothelium”). The immunofluorescence images were carefully examined to detect intercellular gaps and quantify their number and surface area using ImageJ. The gaps were identified as areas of negligible fluorescence intensity between adjacent cells. We found out, however, that after RBC treatment, the cells along the sidewalls of the channel were particularly prone to detachment during the peeling of the channel slab from the bottom substrate prior to immunostaining. Areas of denudation generated by this undesirable cell detachment were excluded from our analysis by calculating the fraction of the area of intercellular gaps in the total surface area of intact cell layer that had not been affected by the peel-off process.

### Mechanical stretching of the endothelium during RBC transfusion

The microvessels in the alveolar regions of the human lung experience cyclic tissue deformation due to breathing motions^31, 41, 53^. To investigate the influence of this physiological biomechanical microenvironment on transfusion-induced endothelial injury, we designed a mechanical stretching system that allowed for computer-controlled application of well-defined cyclic tensile strain to our cell culture microdevice. This platform was realized by mounting motorized linear actuators and device grippers on an acrylic plate (Fig. S1). Briefly, the acrylic plate (40 cm × 25 cm: width × height) was designed to fit on a shelf in a regular cell culture incubator and on a microscope stage. A square opening of 12 cm × 12 cm was cut through the plate to create an open window that could be used for real-time imaging. Two motorized linear stages with built-in controllers (LSM025A, Zaber Technologies, Vancouver, BC, Canada) were fixed on both sides of the acrylic plate, and the custom-machined metal grippers were installed on top of each moving actuator. Each gripper consisted of a long arm with two parallel metal plates attached at the end for holding a microdevice. To integrate our cell culture channel into the stretching system, a fully assembled device was sandwiched between the two metal plates and tightened on both sides with screws. The grippers were then digitally controlled by a LabVIEW interface to laterally stretch and relax our microfluidic device.

During the transfusion experiments, the entire stretching platform was placed in a cell culture incubator maintained at 37 °C and 5% CO2. To mimic physiological breathing conditions^41^, 10% uniaxial tensile strain was applied at 0.2 Hz during the entire duration of transfusion experiments. Concurrently, the cultured cells were exposed to continuous flow of RBC-containing medium at 250 μL/h. Perfusate was collected from the outlet of the device every hour to analyze extracellular HMGB1 released by the endothelial cells.

### Statistical analysis

Statistical significance of the obtained data was evaluated by a two-tailed t-test. Data were presented as the mean ± SEM.

## Electronic supplementary material

Supplementary Figure
