# Supplementary material for: A microengineered model of RBC transfusion-induced pulmonary vascular injury
Source: Sci Rep. 2017 Jun 13;7:3413. doi: 10.1038/s41598-017-03597-w (PMC5469736; doi:10.1038/s41598-017-03597-w)
Supplement: Supplementary file 1 — Supplementary Figure [file 41598_2017_3597_MOESM1_ESM.pdf]

A microengineered model of RBC transfusion-induced pulmonary vascular injury

Jeongyun Seo[1¶], David Conegliano[1¶], Megan Farrell[1], Minseon Cho[1], Xueting Ding[1], Thomas Seykora[1], Danielle Qing[2], Nilam S. Mangalmurti[2], Dongeun Huh[1*]

[1]*Department of Bioengineering, University of Pennsylvania, Philadelphia, PA 19104, USA*

[2]*Pulmonary, Allergy and Critical Care Division, Perelman School of Medicine, Philadelphia, PA 19104, USA*

¶ These authors contributed equally to this work.

*To whom correspondence should be addressed:

E-mail: huhd@seas.upenn.edu, Tel: 1-215-898-5208

Supplementary Figure

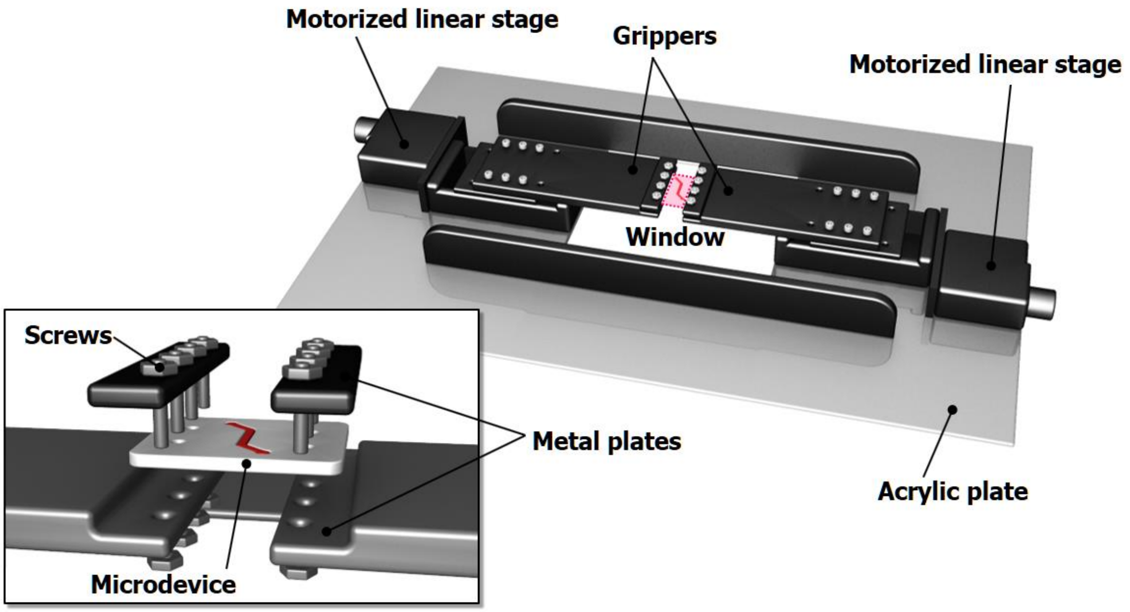

Supplementary Figure 1. A schematic of mechanical stretching system. The cell stretching system consists of two motorized linear stages and metal grippers on an acrylic plate that has an open optical window. For cell stretching, a microfluidic cell culture device is fixed between two metal plates and pulled in the lateral direction by the action of the motorized stages. The magnitude and frequency of stretching can be controlled in a programmable fashion by using a computer equipped with a LabView interface.
